# Supplementary material for: Inhibition of Fungal Strains Isolated from Cereal Grains via Vapor Phase of Essential Oils
Source: Molecules. 2021 Mar 1;26(5):1313. doi: 10.3390/molecules26051313 (PMC7957489; doi:10.3390/molecules26051313)
Supplement: Supplementary file 1 [file molecules-26-01313-s001.zip › SUPPLEMENTARY-Table S2 A heat map of EOs efficiency at 62,5 μL L against fungal strains.pdf]

Table S2: A heat map of EOs efficiency at 62.5 µL/L against fungal strains

**T**	1	2	3	4	5	6	7	8	9	10	11	12	13	14	15	16	17
PO	0.00	0.00	0.67	0.95	1.15	1.23	1.40	1.50	1.62	1.75	1.92	2.07	2.23	2.43	2.63	2.80	2.83
FSP	0.00	0.00	0.00	0.00	0.20	0.55	0.60	0.97	1.23	1.78	2.62	3.30	4.05	4.40	4.50	4.50	4.53
FSO	0.00	0.00	0.00	0.00	0.23	0.57	0.67	0.80	1.03	1.43	2.22	2.68	3.45	3.93	4.07	4.07	4.07
AN	0.20	0.67	1.03	1.70	2.40	2.88	3.32	3.70	4.03	4.17	4.52	4.70	4.75	4.75	4.75	4.75	4.75
AF	0.00	0.78	1.03	1.52	2.17	2.87	3.58	3.83	3.97	4.23	4.58	4.70	4.75	4.75	4.75	4.75	4.75
**O**	1	2	3	4	5	6	7	8	9	10	11	12	13	14	15	16	17
PO	0.00	0.00	0.92	1.23	1.30	1.45	1.60	1.80	1.87	2.03	2.10	2.17	2.20	2.22	2.38	2.48	2.58
FSP	0.00	0.00	0.23	0.25	0.30	0.38	0.47	0.63	1.00	1.93	2.82	3.58	4.30	4.30	4.70	4.75	4.75
FSO	0.00	0.00	0.00	0.00	0.00	0.20	0.53	0.87	1.43	2.52	3.35	4.03	4.40	4.40	4.63	4.75	4.75
AN	0.20	1.00	1.72	2.40	3.08	3.67	4.03	4.20	4.42	4.75	4.75	4.75	4.75	4.75	4.75	4.75	4.75
AF	0.00	0.77	1.35	2.12	3.02	3.52	4.00	4.33	4.58	4.75	4.75	4.75	4.75	4.75	4.75	4.75	4.75
**L**	1	2	3	4	5	6	7	8	9	10	11	12	13	14	15	16	17
PO	0.00	0.00	0.45	0.93	1.20	1.78	2.43	2.80	2.87	2.93	2.93	2.93	2.93	2.93	2.93	2.93	2.93
FSP	0.00	0.13	0.95	2.20	3.72	4.75	4.75	4.75	4.75	4.75	4.75	4.75	4.75	4.75	4.75	4.75	4.75
FSO	0.00	0.13	1.07	2.73	4.32	4.75	4.75	4.75	4.75	4.75	4.75	4.75	4.75	4.75	4.75	4.75	4.75
AN	0.00	0.23	1.45	2.95	4.07	4.75	4.75	4.75	4.75	4.75	4.75	4.75	4.75	4.75	4.75	4.75	4.75
AF	0.00	1.07	2.73	3.90	4.33	4.63	4.63	4.75	4.75	4.75	4.75	4.75	4.75	4.75	4.75	4.75	4.75
**CL**	1	2	3	4	5	6	7	8	9	10	11	12	13	14	15	16	17
PO	0.33	1.20	1.57	1.83	2.10	2.47	2.70	2.93	2.95	2.97	2.97	2.97	2.97	2.97	2.97	2.97	2.97
FSP	0.48	1.87	2.87	3.95	4.42	4.75	4.75	4.75	4.75	4.75	4.75	4.75	4.75	4.75	4.75	4.75	4.75
FSO	0.72	2.80	4.03	4.75	4.75	4.75	4.75	4.75	4.75	4.75	4.75	4.75	4.75	4.75	4.75	4.75	4.75
AN	0.73	2.00	2.67	3.20	3.77	4.12	4.50	4.63	4.75	4.75	4.75	4.75	4.75	4.75	4.75	4.75	4.75
AF	0.92	1.92	2.88	3.50	3.97	4.07	4.50	4.60	4.67	4.75	4.75	4.75	4.75	4.75	4.75	4.75	4.75
**Control**	1	2	3	4	5	6	7	8	9	10	11	12	13	14	15	16	17
PO	0.96	1.69	2.45	3.07	3.56	3.76	4.05	4.15	4.20	4.20	4.20	4.20	4.20	4.20	4.20	4.20	4.20
FSP	1.05	2.84	4.72	4.75	4.75	4.75	4.75	4.75	4.75	4.75	4.75	4.75	4.75	4.75	4.75	4.75	4.75
FSO	1.09	3.05	4.70	4.75	4.75	4.75	4.75	4.75	4.75	4.75	4.75	4.75	4.75	4.75	4.75	4.75	4.75
AN	1.25	2.64	4.25	4.45	4.59	4.75	4.75	4.75	4.75	4.75	4.75	4.75	4.75	4.75	4.75	4.75	4.75
AF	1.19	2.53	3.72	4.36	4.65	4.75	4.75	4.75	4.75	4.75	4.75	4.75	4.75	4.75	4.75	4.75	4.75

PO = *Penicillium ochrochloron*; FSP = *Fusarium sporotrichioides*; FSO = *Fusarium solani*; AN = *Aspergillus niger*; AF = *Aspergillus flavus*; T = thyme; O = oregano; L = lemongrass; CL = clove.

The heat map shows graphical representation of fungal growth (cm) over 17 days after treatment with different EOs vapors at 62.5 µL/L.
