# Supplementary material for: Inhibition of Fungal Strains Isolated from Cereal Grains via Vapor Phase of Essential Oils
Source: Molecules. 2021 Mar 1;26(5):1313. doi: 10.3390/molecules26051313 (PMC7957489; doi:10.3390/molecules26051313)
Supplement: Supplementary file 1 [file molecules-26-01313-s001.zip › SUPPLEMENTARY-Table S1 Relative fungal growth after EOs treatment at 62.5 μL L.pdf]

**Table S1:** Relative fungal growth after EOs treatment at 62.5 μL/L

| **PO** | 1 | 2 | 3 | 4 | 5 | 6 | 7 | 8 | 9 | 10 | 11 | 12 | 13 | 14 | 15 | 16 | 17 |
|---|---|---|---|---|---|---|---|---|---|---|---|---|---|---|---|---|---|
| T | 0.00 a | 0.00 a | 27.21 abcd | 30.94 bc | 32.30 bc | 32.80 bc | 34.57 bc | 36.14 ab | 38.49 ab | 41.67 a | 45.63 a | 49.21 a | 53.17 a | 57.94 a | 62.70 a | 66.67 ab | 67.46 a |
| O | 0.00 a | 0.00 a | 37.41 abcde | 40.17 cd | 36.52 c | 38.56 cd | 39.51 cd | 43.37 b | 44.44 abc | 48.41 ab | 50.00 a | 51.59 a | 52.38 a | 52.78 a | 56.75 a | 59.13 a | 61.51 a |
| CL | 34.72 ab | 71.01 fg | 63.95 defg | 60.31 cdefg | 49.55 cd | 65.60 efg | 66.67 ef | 70.68 c | 70.24 cde | 70.63 abc | 70.63 abcd | 70.63 ab | 70.63 ab | 70.63 ab | 70.63 ab | 70.63 abc | 70.63 ab |
| L | 0.00 a | 0.00 a | 18.37 ab | 30.40 bc | 33.71 c | 47.43 cde | 60.08 de | 67.47 c | 68.25 bcd | 69.84 abc | 69.84 abcd | 69.84 ab | 69.84 ab | 69.84 ab | 69.84 ab | 69.84 ab | 69.84 ab |
| **FSP** | | | | | | | | | | | | | | | | | |
| T | 0.00 a | 0.00 a | 0.00 a | 0.00 a | 4.21 a | 11.58 ab | 12.63 ab | 20.35 ab | 25.96 a | 37.54 a | 55.09 ab | 69.47 ab | 85.26 bc | 92.63 bc | 94.74 c | 94.74 cd | 95.44 bc |
| O | 0.00 a | 0.00 a | 4.94 ab | 5.26 ab | 6.32 ab | 8.07 ab | 9.82 a | 13.33 a | 21.05 a | 40.70 a | 59.30 abc | 80.70 ab | 90.53 bc | 98.25 c | 98.95 c | 100.00 d | 100.00 c |
| CL | 46.03 abc | 65.73 efg | 60.73 cdefg | 83.16 fgh | 92.98 gh | 100.00 hi | 100.00 h | 100.00 de | 100.00 ef | 100.00 c | 100.00 d | 100.00 b | 100.00 c | 100.00 c | 100.00 c | 100.00 d | 100.00 c |
| L | 0.00 a | 4.69 ab | 20.13 ab | 46.32 cde | 78.25 efgh | 100.00 hi | 100.00 h | 100.00 de | 100.00 ef | 100.00 c | 100.00 d | 100.00 b | 100.00 c | 100.00 c | 100.0 c | 100.00 d | 100.00 c |
| **FSO** | | | | | | | | | | | | | | | | | |
| T | 0.00 a | 0.00 a | 0.00 a | 0.00 a | 4.91 a | 11.93 ab | 14.04 ab | 16.84 a | 21.75 a | 30.18 a | 46.67 a | 56.49 a | 72.63 ab | 82.81 bc | 85.61 bc | 85.61 bcd | 85.61 abc |
| O | 0.00 a | 0.00 a | 0.00 a | 0.00 a | 0.00 a | 4.21 a | 11.23 a | 18.25 a | 32.63 a | 52.98 ab | 70.53 abcd | 84.91 ab | 92.63 bc | 96.14 c | 97.54 c | 98.25 d | 100.00 c |
| CL | 65.75 bc | 91.80 g | 85.82 gh | 100.00 h | 100.00 h | 100.00 hi | 100.00 h | 100.00 de | 100.00 ef | 100.00 c | 100.00 d | 100.00 b | 100.00 c | 100.00 c | 100.00 c | 100.00 d | 100.00 c |
| L | 0.00 a | 4.37 ab | 22.7ab | 57.54 cdef | 90.88 fgh | 100.00 hi | 100.00 h | 100.00 de | 100.00 ef | 100.00 c | 100.00 d | 100.00 b | 100.00 c | 100.00 c | 100.00 c | 100.00 d | 100.00 c |
| **AN** | | | | | | | | | | | | | | | | | |
| T | 16.00 ab | 23.36 abcd | 24.31 abc | 38.20 cd | 52.29 cde | 60.70 defg | 69.82 ef | 77.89 cd | 84.91 def | 87.72 bc | 95.09 bcd | 98.95 b | 100.00 c | 100.00 c | 100.00 c | 100.00 d | 100.00 c |
| O | 16.00 ab | 37.88 cde | 40.39 bcdef | 53.93 cdef | 67.18 defg | 77.19 fghi | 84.91 fgh | 88.42 cde | 95.44 def | 100.00 c | 100.00 d | 100.00 b | 100.00 c | 100.00 c | 100.00 c | 100.00 d | 100.00 c |
| CL | 58.67 abc | 75.76 g | 62.75 defg | 71.91 efgh | 82.06 fgh | 86.67 ghi | 94.74 gh | 97.54 de | 100.00 ef | 100.00 c | 100.00 d | 100.00 b | 100.00 c | 100.00 c | 100.00 c | 100.00 d | 100.00 c |
| L | 0.00 a | 8.84 abc | 34.12 abcd | 66.29 defg | 88.60 fgh | 100.00 hi | 100.00 h | 100.00 de | 100.00 ef | 100.00 c | 100.00 d | 100.00 b | 100.00 c | 100.00 c | 100.00 c | 100.00 d | 100.00 c |
| **AF** | | | | | | | | | | | | | | | | | |
| T | 0.00 a | 33.60 bcd | 27.78 abcd | 34.79 bc | 46.59 cd | 60.35 def | 75.44 efg | 80.70 cde | 83.51 def | 89.12 bc | 96.49 cd | 98.95 b | 100.00 c | 100.00 c | 100.00 c | 100.00 d | 100.00 c |
| O | 0.00 a | 30.30 abcd | 36.29 abcde | 48.55 cde | 64.87 def | 74.04 fgh | 84.21 fgh | 88.42 cde | 98.25 def | 100.00 c | 100.00 d | 100.00 b | 100.00 c | 100.00 c | 100.00 c | 100.00 d | 100.00 c |
| CL | 77.03 bc | 75.76 g | 77.51 fgh | 80.28 fgh | 85.30 fgh | 85.61 fghi | 94.74 gh | 96.84 de | 98.25 def | 100.00 c | 100.00 d | 100.00 b | 100.00 c | 100.00 c | 100.00 c | 100.00 d | 100.00 c |
| L | 0.00 a | 42.16 def | 73.48 efgh | 89.45 gh | 93.19 gh | 97.54 hi | 100.00 h | 100.00 de | 100.00 ef | 100.00 c | 100.00 d | 100.00 b | 100.00 c | 100.00 c | 100.00 c | 100.00 d | 100.00 c |
| **Control** | | | | | | | | | | | | | | | | | |
| PO | 100.00 c | 100.00 h | 100.00 h | 100.00 h | 100.00 h | 100.00 i | 100.00 h | 100.00 e | 100.00 f | 100.00 c | 100.00 d | 100.00 b | 100.00 c | 100.00 c | 100.00 c | 100.00 d | 100.00 c |
| FSP | 100.00 c | 100.00 h | 100.00 h | 100.00 h | 100.00 h | 100.00 i | 100.00 h | 100.00 e | 100.00 f | 100.00 c | 100.00 d | 100.00 b | 100.00 c | 100.00 c | 100.00 c | 100.00 d | 100.00 c |
| FSO | 100.00 c | 100.00 h | 100.00 h | 100.00 h | 100.00 h | 100.00 i | 100.00 h | 100.00 e | 100.00 f | 100.00 c | 100.00 d | 100.00 b | 100.00 c | 100.00 c | 100.00 c | 100.00 d | 100.00 c |
| AN | 100.00 c | 100.00 h | 100.00 h | 100.00 h | 100.00 h | 100.00 i | 100.00 h | 100.00 e | 100.00 f | 100.00 c | 100.00 d | 100.00 b | 100.00 c | 100.00 c | 100.00 c | 100.00 d | 100.00 c |
| AF | 100.00 c | 100.00 h | 100.00 h | 100.00 h | 100.00 h | 100.00 i | 100.00 h | 100.00 e | 100.00 f | 100.00 c | 100.00 d | 100.00 b | 100.00 c | 100.00 c | 100.00 c | 100.00 d | 100.00 c |

PO = *Penicillium ochrochloron*; FSP = *Fusarium sporotrichioides*; FSO = *Fusarium solani*; AN = *Aspergillus niger*; AF = *Aspergillus flavus*; T = thyme; O = oregano; CL = clove; L = lemongrass
The table represents comparison between effect of EOs at 62.5 μL/L against different fungi over 17 days. The data were normalized to a percentage fungal growth each day, with the growth in control that day equalling 100%. The letters in columns signify cases with no statistical differences ($p > 0.05$) between samples according the Scheffe´s test.
